# Porcine Lymphotropic Herpesviruses (PLHVs) and Xenotranplantation

**DOI:** 10.3390/v13061072

**Published:** 2021-06-04

**Authors:** Joachim Denner

**Affiliations:** Institute of Virology, Free University, 14163 Berlin, Germany; Joachim.Denner@fu-berlin.de; Tel.: +49-30-838-63059

**Keywords:** porcine lymphotropic herpesviruses, xenotransplantation, porcine cytomegalovirus

## Abstract

Porcine lymphotropic herpesviruses -1, -2 and -3 (PLHV-1, PLHV-2 and PLHV-3) are gammaherpesviruses which are widespread in pigs. They are closely related to the Epstein–Barr virus (EBV) and Kaposi sarcoma herpesvirus, both of which cause severe diseases in humans. PLHVs are also related to bovine and ovine gammaherpesviruses, which are apathogenic in the natural host, but cause severe diseases after transmission into other species. Until now, no association between PLHVs and any pig diseases had been described. However, PLHV-1 causes a post-transplantation lymphoproliferative disorder (PTLD) after experimental transplantations in minipigs. This disorder is similar to human PTLD, a serious complication of solid human organ transplantation linked to EBV. Xenotransplantation using pig cells, tissues and organs is under development in order to alleviate the shortage of human transplants. Meanwhile, remarkable survival times of pig xenotransplants in non-human primates have been achieved. In these preclinical trials, another pig herpesvirus, the porcine cytomegalovirus (PCMV), a roseolovirus, was shown to significantly reduce the survival time of pig xenotransplants in baboons and other non-human primates. Although PLHV-1 was found in genetically modified donor pigs used in preclinical xenotransplantation, it was, in contrast to PCMV, not transmitted to the recipient. Nevertheless, it seems important to use PLHV-free donor pigs in order to achieve safe xenotransplantation.

## 1. Xenotransplantation: Progress and Virus Safety

Xenotransplantation using pig cells, tissues and organs is under development in order to alleviate the lack of human transplants. For example, in the USA 107,000 men, women, and children are on the national transplant waiting list, as of February 2021. However, only 39,000 transplants were performed in 2020 and 17 people die each day waiting for an organ transplant [1]. To alleviate the shortage of human organs, xenotransplantation is under development. For several reasons, pigs are the most suited donor animals and rejection of pig xenotransplants will be prevented by genetic modifications of the animals [2]. Thanks to these genetic modifications and new powerful immunosuppressive regimens, remarkable survival times have been achieved in preclinical trials with non-human primates [3,4]. For example, pig islet cells for the treatment of diabetes survived 950 days [5] and pig hearts transplanted heterotopically survived 945 days [6]. Whereas in 2011, the survival time of orthotopic (life-supporting) heart transplants was 57 days [7], but thanks to a better strategy, the survival time was 192 days in 2020 [8] and 9 months in 2021 [9]. This improved experimental strategy also included screening for and eliminating the porcine cytomegalovirus (PCMV), which is actually a porcine roseolovirus (PCMV/PRV) [10]. It has been shown in numerous preclinical trials that the presence of PCMV/PRV in the pig organ significantly decreases the survival time of the xenotransplant in non-human primates (for review see [11]). PCMV/PRV, also called suid herpesvirus 2 (SuHV-2), is ubiquitous and may cause foetal or neonatal deaths in pigs and it has been associated with runting, rhinitis and pneumonia in piglets. PCMV/PRV was repeatedly found transmitted with pig kidneys and heart transplantations to non-human primates, which were always associated with a significant reduction in the survival time of the xenotransplant. Transmission of PCMV/PRV reduced the survival time of pig kidneys, on average, from 53 days to 14 days in baboons [12], from 28 to 9 days in cynomolgus monkeys [13] and reduced the survival of orthotopically transplanted pig hearts in baboons from 195 days to less than 30 days [14]. These studies also showed for the first time that PCMV/PRV is obviously able to decrease the survival time of the pig transplant without infecting the host, modulating cytokine release and coagulation by interactions with the immune system and endothelial cells [14].

These data show that the transmission of potentially zoonotic viruses from the donor pig to the recipient, and in the near future to human patients, may pose a significant risk for the recipient and therefore donor animals without zoonotic viruses, including herpesviruses, should be used.

## 2. PLHVs: Biology and Prevalence

Herpesviruses (family Herpesviridae) are pathogens that infect a wide variety of animals, including humans, monkeys, birds, frogs, and fish. The Herpesviridae family contains three subfamilies: alphaherpesvirinae, betaherpesvirinae, and gammaherpesvirinae. Nine human herpesviruses (HHV) have been identified that cause a wide spectrum of human diseases: herpes simplex virus 1 (HSV-1) (HHV-1) causes herpes genitalis and labialis, encephalitis, vision loss and others; HSV-2 (HHV-2)—genital herpes; varicella zoster virus (VZV) (HHV-3)—chickenpox and shingles; Epstein–Barr virus (EBV) (HHV-4)—infectious mononucleosis; cytomegalovirus (CMV) (HHV-5)—mononucleosis and pneumonia; HHV-6A and HHV-6B are the causative agents of roseola (exanthema subitum); HHV-7 is associated with several syndromes, including exanthema subitum and the Kaposi sarcoma herpesvirus is HHV-8.

During the course of evolution, most herpesviruses have adapted to a single or a limited number of host species. However, herpesviruses are not so species-specific as previously thought [15], and in recent years, numerous trans-species transmissions of several members of the Herpesviridae family have been reported, which have often resulted in severe or fatal outcomes in the new hosts [16]. For example, herpes B virus is a zoonotic herpesvirus that infects macaques and causes approximately 80% mortality in untreated humans [17].

In pigs, suid herpesvirus 1 (SuHV-1), also known as pseudorabies virus (PRV), causes Aujeszky’s disease in both wild and domestic swine. SuHV-1 has a broad host range and causes disease in distantly related mammals such as sheep, dogs, cattle, mink, and puma [18,19]. SuHV-2 is the above mentioned PCMV/PRV [10].

Porcine lymphotropic herpesviruses -1, -2, and -3 (PLHV-1, PLHV-2, and PLHV-3), also known as SuHV-3, SuHV-4, and SuHV-5, are gammaherpesviruses assigned to the genus Macavirus. Their role as primary pathogens of swine, or as co-factors in other viral infections, is largely unknown. They were found in domestic and wild pig populations and their frequency is comparable between healthy and diseased animals. The prevalence of PLHV-1, PLHV-2, and PLHV-3 varies between 29% and 80%, between 11% and 41%, and between 5 and 65%, respectively, in various studies performed in Germany, Italy, Spain, France, USA, and Ireland, and their prevalence has not changed significantly in the last 20 years [20,21,22,23]. Despite their high prevalence, PLHVs’ relevance for the swine industry appears low. Their transmission occurs mainly horizontally, but vertical transmission is possible [24,25,26]. PLHVs were found in different pig tissues and in the persistently infected porcine B cell line L23 [20,27]. PLHV-1 causes a post-transplantation lymphoproliferative disorder (PTLD) in minipigs after experimental allogenic bone marrow transplantations [28,29,30]. This disorder is similar to human PTLD, a serious complication of solid human organ transplantation linked to EBV (HHV-4).

Porcine lymphotropic herpesviruses (PLHVs) are closely related to alcelaphine herpesvirus type 1, AlHV-1, and ovine herpesvirus type 2, OvHV-2, two gammaherpesviruses. These viruses are apathogenic in their natural hosts but cause serious lymphoproliferative diseases in other species [31]. Accordingly, PLHV could be apathogenic in pigs but pathogenic in other species.

## 3. Detection Methods

To detect PLHVs, PCR tests and to quantify them, real-time PCRs have been used [20,22,23,24,25,26,27,28,29,30,32,33,34,35,36,37,38,39,40,41,42]. The primers and probes were located in the DNA polymerase gene and in the gene encoding the glycoprotein B.

To detect an antibody response in the infected animals, a Western blot assay and ELISA were established using the recombinant glycoprotein B [43,44]. Antibodies were found in slaughterhouse pigs, which were all PLHV-3 positive by PCR, but not in Göttingen minipigs [44].

## 4. No Transmission of PLHV in Preclinical and Clinical Xenotransplantation Trials

As described above, PLHVs are very common in slaughterhouse pigs, but they were also detected in pigs generated for xenotransplantation, e.g., in miniature swine and large white pigs [25]. Although caesarean derivation and barrier maintenance significantly reduced the incidence of PLHV infection, in comparison with conventionally reared pigs (from 80% down to 3 to 12.8%), it was not sufficient to eliminate the virus [25]. PLHVs were not found in Göttingen minipigs [35,36,44], which will be used for islet cell transplantation in a German trial and were consequently not transmitted when islet cells of these animals were transplanted in a preclinical trial to rhesus monkeys [37]. In a large study, piglets with the genetic background Landrace x Yorkshire, obtained by somatic cell nuclear transfer (SCNT) and derived via caesarean section, were analysed for 30 different porcine viruses using real-time PCR methods [26]. PLHV-3 was detected in five of nine and PLHV-2 in three of nine piglets, whereas no other viruses were found, with the exception of influenza B virus. This study shows that caesarean section can remove PLHVs but is not 100% safe. In another large study, large White-Yorkshire x Landrace F1 animals, used as pancreas donor animals, were screened for more than 30 known viruses [38]. Islets cells from these animals were transplanted into cynomolgus monkeys and none of the donor pigs and recipients had PLHV-1, -2 or -3. In another study, pig kidneys and hearts were transplanted into immunosuppressed baboons [39]. All donor animals carried PCMV, and 55% of them carried PLHV. An increased expression (activation) of PCMV was detected in all recipients, but in contrast to PCMV and despite immunosuppression and transplant rejection, neither transplants that were carrying PLHV-1, nor those that were negative, developed an increased expression (activation) of PLHV-1 [39]. Replication of PLHV was not observed in the baboon recipient despite prolonged pig cell microchimerism [40].

Although PLHV was found in eight out of eight genetically modified pigs used for orthotopic pig heart transplantation, it was not detected in the baboon recipients [14]. PLHVs were not transmitted in the first clinical trials transplanting encapsulated islet cells from Auckland Island pigs, because the donor animals were PLHV negative [41,42].

Reactivation of latent herpesviruses is an important cause of morbidity and mortality in human transplantation. It remains unclear whether zoonotic pig viruses could be reactivated in the transplanted tissue and interact with related human viruses. PLHV-1 encodes several genes with a strong transactivating effect on virus reactivation and replication, which are conserved in gammaherpesviruses, and it was shown that PLHV-1 transactivators upregulated HHV-8 and EBV promoters [45], supporting the hypothesis that PLHV-1 might have pathogenic relevance in the course of xenotransplantation.

## 5. Treatment, Vaccination and Elimination

At present, there is no treatment and no vaccination against all three PLHVs. In addition, other methods such as early weaning and colostrum deprivation, failed [24], although they had been successfully used in the case of PCMV [24,46]. Unfortunately, caesarean derivation was only partially successful [24,25,26]. Therefore, most of the elimination programs proposed for other viruses [47] cannot be used for PLHV. Only caesarean derivation and selection of the negative animals using sensitive detection methods described above will be successful and can be applied to eliminate PLHV from donor pigs if necessary.

## 6. Conclusions

Evidence has accumulated that herpesviruses are not as species-specific as thought in the past and that trans-species transmissions of members of the Herpesviridae family resulted in severe or fatal outcomes in the new host. On the other hand, although PLHV was found in genetically modified donor pigs used in preclinical xenotransplantations, it was not, in contrast to PCMV, transmitted to the recipients. Nevertheless, it seems important to use PLHV-free pigs in order to achieve safe xenotransplantation.
